# A mixed methods exploration of motor imagery in autistic and non-autistic adults: Diverse experiences and implications for interventions

**DOI:** 10.1371/journal.pone.0326542

**Published:** 2025-06-26

**Authors:** Molly Brillinger, Ying Bai, April Karlinsky, Timothy N. Welsh, Ellen Poliakoff, Emma Gowen

**Affiliations:** 1 Faculty of Kinesiology & Physical Education, University of Toronto, Toronto, Ontario, Canada; 2 Division of Psychology, Communication and Human Neuroscience, School of Health Sciences, Faculty of Biology, Medicine and Health, University of Manchester, Academic Health Science Centre, Manchester, United Kingdom; 3 Department of Kinesiology, California State University-San Bernardino, San Bernardino, California, United States of America; University of Illinois Urbana-Champaign, UNITED STATES OF AMERICA

## Abstract

Research on motor imagery (MI) in non-autistic individuals suggests that there are shared neural circuitries between imagery and execution. The relationship between imagined and executed movements, and the use of MI in autistic adults is poorly understood. This study explored MI comprehension, prior use of MI, and subjective experiences during MI in autistic and non-autistic adults. Twenty autistic and twenty non-autistic individuals responded to a series of questions probing their understanding of and engagement in MI. Participants then completed the Kinesthetic and Visual Imagery Questionnaire (KVIQ), and reported on their subjective experiences during MI. Although there were no differences between the autistic and non-autistic individuals in their understanding of MI, the non-autistic group may have more prior use of MI in their everyday lives. Additionally, autistic participants generally reported less vivid imagery on the KVIQ compared to non-autistic participants, however experiences during MI varied widely across both groups ranging from vivid/intense images/sensations to the inability to imagine. In summary, some autistic individuals are able to engage in MI, but, similar to their non-autistic peers, MI ability and experiences vary across individuals. This work has important implications for MI interventions aimed at improving motor coordination.

## Introduction

Autism spectrum condition is a neurodevelopmental condition characterized by differences in social communication and repetitive, stereotyped behaviours [1]. Although not considered a defining trait of autism, motor coordination difficulties are also prevalent whereby a large proportion of autistic individuals (~80%) experience challenges with fine and gross motor control, postural control, and hand-eye coordination [2–4]. Because movement is fundamental to daily life, motor-related challenges can negatively impact many domains of an individual’s life including everyday living skills (e.g., cooking, tying shoes), mental health, and general quality of life [5]. As such, researchers have begun recognizing motor-related challenges as a potentially important aspect of autism by investigating the underlying mechanisms [6,7], potential diagnostic patterns [8,9], and possible intervention methods [10].

There is also interest in understanding how these motor challenges might relate to differences in social communication in autism [11,12, for a review, see 13]. The common coding theory is based on the premise that the neural codes leading to the generation of action are tightly linked or coupled to the codes that represent the perceptual effects or outcomes of the action. These coupled perception-action codes are not only engaged when executing an action, but also during action perception and imagination (albeit at a sub-threshold level). Thus, there is proposed to be a shared neural coding system for action execution, perception, and imagination processes. In the context of perception, watching an action and/or perceiving the effects of the action prompts an internal simulation of the action in the observer, allowing them to understand, predict, or even imitate the actions [14].

Motor imagery (MI) is a specialized form of motor simulation that involves an individual imagining performing a movement, without physically moving. Previous research in the general population has unveiled important behavioural and neurophysiological similarities between MI and physical execution of an action [for reviews, see 15,16]. For instance, the time it takes to imagine performing a particular movement (i.e., imagined movement time) is similar to the time it takes to physically perform that same movement ( [17–19]. Neuroimaging studies have also identified overlapping (but not identical) neural activation patterns between MI and physical execution, centering around the supplementary motor area, premotor cortex, and sensorimotor cortex [for reviews, see 16,20]. Together, the evidence implies a strong correspondence between imagined and physically executed movements, lending support for the common coding network underlying these motor-cognitive processes.

Considering the prevalence of motor coordination difficulties in autism and the common codes on which these processes may operate, it is possible that autistic individuals have challenges in MI as well. In other words, for autistic individuals, any difficulties experienced during physical execution of a movement may be mirrored when imagining themselves performing that same movement, and/or they may have difficulty producing MI itself. Previous research has shown alterations in some behaviours that involve simulation such as action prediction [e.g., 21,22], action understanding [e.g., 23], and imitation processes [e.g., 24–26] in autism. Less is known about MI processes in autism [27]. The current study was designed to examine motor-related processes in autistic individuals, with a specific focus on the understudied process of MI.

Much of the literature examining MI in autistic individuals focuses on implicit MI, where participants are not directly instructed to engage in MI, but are instead instructed to make judgements based on visual stimuli of certain body parts (e.g., judging the laterality [left or right] of a hand presented at various orientations) – a task that implicitly prompts the mental rotation and simulation of their own body parts [28]. Findings from hand rotation tasks suggest that autistic individuals perform implicit MI, but that autistic individuals may experience more challenges during MI compared to their non-autistic counterparts [29–31]. Evidence from body rotation tasks is limited with few studies and mixed findings [e.g., 32,33]. Notably, these implicit MI studies almost exclusively examined children or adolescents, leaving little understanding of such processes in autistic adults.

The evidence pertaining to explicit MI in autistic adults is also scarce. In explicit MI tasks, participants are overtly instructed to engage in MI either visually, kinesthetically, or both. Visual MI requires an individual to imagine what a particular movement would look like to perform (from a first- and/or a third-person perspective), whereas kinesthetic MI requires an individual to imagine the feelings and sensations associated with performing a particular movement. The few studies examining explicit MI in autistic children using a variety of different tasks report mixed findings of both present [34] and absent [35–37] MI.

In non-autistic populations, questionnaires are commonly used to measure explicit MI and the individual’s own subjective assessment of their ability to engage in explicit MI. The Kinesthetic and Visual Imagery Questionnaire (KVIQ), for example, is an explicit MI measure specifically developed for people with reduced mobility or physical disabilities [38]. The KVIQ provides insight into both visual MI and kinesthetic MI by assessing an individual’s ability to visualize and feel imagined movements, respectively. Within the KVIQ, participants physically perform basic movements (e.g., lift up the non-dominant arm) before imagining themselves performing the same movements. After imagining each movement, participants are asked to rate: 1) the clarity of their visual imagery (visual MI) and 2) the intensity of their imagined sensations (kinesthetic MI). The ratings are provided on separate 5-point scales, both ranging from 1 (‘no image’/ ‘no sensation’) to 5 (‘image as clear as seeing’/ ‘as intense as executing the action’). Only one study has administered the KVIQ to assess explicit MI in autistic adults and showed no significant differences between autistic and non-autistic adults on the visual or kinesthetic subscale [21]. These findings suggest autistic adults have the capacity to engage in explicit MI, and that the self-reported vividness of their imagined movements is comparable to non-autistic individuals. This result contrasts with the findings mentioned earlier suggesting that autistic adults do not have the capacity to engage in explicit MI [35]. Clearly, more research is required to understand possible differences between autistic and non-autistic individuals in explicit MI.

In summary, there is limited knowledge regarding explicit MI in autistic adults. Not only do previous studies present varying results, but the subjective experiences of autistic individuals when engaging in explicit MI have not been examined. The purpose of the present study was to start developing a comprehensive understanding of explicit MI in autistic individuals using a nested qualitative-quantitative mixed-methods approach. The present paper, which was part of a larger project [39], reports an analysis of data from participants who completed an online version of the KVIQ before executing, perceiving, and imagining hand movements in an online manual aiming task that assesses Fitts’ law [see 39 for detailed methods and results from Fitts’ law task]. The qualitative approach may help clarify the differing quantitative findings in the literature by way of identifying explicit MI strategies and abilities which autistic individuals possess. Qualitative data were obtained using open questions asking participants about their understanding of MI and what they experienced when using MI during the KVIQ. Quantitative data were obtained from participants responses on the KVIQ, as well as bespoke closed and open questions probing participants’ knowledge and prior use of MI. We also explored the relationship between motor coordination and MI by correlating KVIQ scores with scores on a motor coordination questionnaire, predicting a negative relationship. Given the novelty of the present mixed-methods approach, it was challenging to form a priori predictions about the level of understanding of MI in autistic and non-autistic individuals. Nonetheless, establishing a more nuanced and comprehensive understanding of explicit MI processes and experiences in autistic adults should offer unique insights into the associated motor coordination difficulties and whether MI could be used as a tool to improve motor coordination.

## Methods

This study is part of a larger study that was pre-registered using the Open Science Framework (http://osf.io/26azs). This study adhered to the GRASS guidelines for reporting MI studies [40; see S4-S6 Tables for checklists].

### Participants

Twenty autistic and twenty non-autistic individuals of similar age, sex, handedness, and full-scale intelligence quotient [FSIQ-2; 41] were recruited through the Body, Eye and Movement (BEAM) laboratory database, the Autism@Manchester mailing list, local support groups, and volunteer advertisements (Table 1). The inclusion criteria required participants to be between 18 and 45 years of age, have normal or corrected-to-normal vision, have no history of neurological movement disorders (e.g., Parkinson’s disease), no diagnosis of a learning disability, and have access to a computer with internet connection, audio capabilities, and a webcam. The sample size was determined to align with the primary objectives of the broader research project. That is, the sample size was based on a previous study using the same motor task (i.e., Fitts’ law task) with a similar research question as well as a power analysis using simulated data (Power >0.99) [19]. All autistic participants had a professional diagnosis of autism, confirmed by a letter of diagnosis, and scored above the cut-offs for difficulties with social reciprocal behaviour on the second edition of the Social Responsiveness Scale [SRS-2; 42]. The SRS-2 is a validated measure of the social impairments of autism and is used widely during comprehensive assessments of autism. A score of 60+ on the SRS-2 indicates clinically significant difficulties with reciprocal social behaviour. Participants in the non-autistic group did not have a clinical diagnosis of autism and scored lower than 60 on the SRS-2, which according to the SRS-2 guidelines is not associated with clinically significant autism. The autistic group scored higher than the non-autistic group on both the SRS-2 and the Adult Developmental Coordination Disorders/Dyspraxia Checklist [ADC; [43] (Table 1). All data were collected between November 25^th^, 2021 and August 8^th^, 2022. All participants provided written informed consent via an online survey platform (Qualtrics; www.qualtrics.com) and the study was approved by the University of Manchester Research Ethics Committee.

**Table 1:** Participant demographics.

	Autistic (*n* = 20)	Non-autistic (*n* = 20)	Group comparison
Age	29.30 years (7.66)	28.95 years (6.44)	*t* (38) = 0.16, *p* = 0.88
Sex	15 Female	13 Female	*X*^2^ (1, N = 40) = 0.48, *p* = 0.49
Handedness	17 right-handed	19 right-handed	*X*^2^ (1, N = 40) = 0.28, *p* = 0.60
FSIQ-2	120.75 (13.74)	113.00 (14.56)	*t* (38) = 1.73, *p* = 0.09
SRS-2^*^	110.95 (21.57)	44.50 (15.65)	*t* (38) = 11.15, *p < *0.001
ADC^*^	93.10 (14.82)	65.20 (10.36)	*t* (38) = 6.90, *p < *0.001

Mean (and standard deviation) values for the Full Scale IQ with 2 subtest score (FSIQ-2: Vocabulary and Matrix Reasoning), Social Responsiveness Scale (Second Edition; SRS-2), and Adult Developmental Coordination Disorder/Dyspraxia Checklist (ADC). An asterisk (*) represents a significant between-group difference.

### Study procedure and measures

The present study examined data from questions about MI completed by participants as part of a larger project examining explicit MI [39]. For the sake of brevity, only the measures and research questions relevant to the current research questions are described and reported herein. Data for the present study were collected during the COVID-19 pandemic and, as such, participants completed the study online on their personal computer, in a location of their choice. At the beginning of the session, participants were asked to sit in front of a computer in a quiet room. They received a Zoom meeting invitation from the experimenter (YB) where they were encouraged to enable both audio and video for the entire session which all participants did. The purpose of the video call was to monitor the participant’s progress and provide support throughout the session, rather than to collect and/or review data. The experimenter first provided an overview of the study and provided a link for the participant to give their electronic informed consent. During this video call, participants were encouraged to ask questions or express any concerns to the researcher regarding the study, to ensure all participants understood the implications of their involvement before providing consent. Ahead of the session, participants were also given a Participant Information Sheet, a sample consent form, and a more detailed “What to Expect” document outlining what would happen during the study, which autistic people have confirmed as being helpful for preparing. Demographic data including age, sex, and educational background were collected via the Qualtrics online platform (www.qualtrics.com), as well as the Edinburgh Laterality Inventory to determine handedness [44] (Table 1).

**Fig 1:**
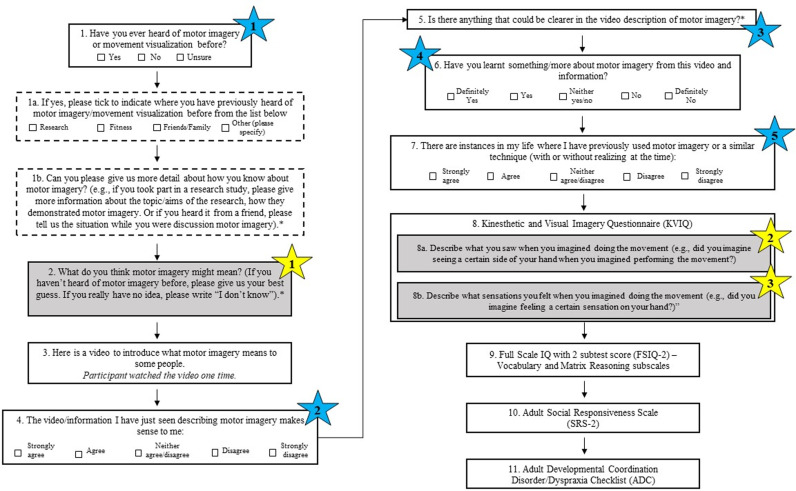
Flowchart of Participant Question Order. Flowchart demonstrating order of bespoke questions regarding participants’ knowledge, understanding, and prior use of MI [1–7] and published questionnaires [8–11] (KVIQ, FSIQ-2, SRS-2, ADC). Participants answered all questions online via the Qualtrics survey platform (www.qualtrics.com). Blue stars marked 1-5 indicate the five questions that were analyzed to examine participants’ knowledge and prior use of MI, as well as participants’ understanding of MI concepts presented in the informational video. Yellow stars with shaded boxes indicate the three questions for which their data were thematically analysed. An asterisk (*) indicates a question in which participants provided a typed response. Dashed boxes indicate questions that participants answered only if they responded ‘Yes’ to question #1. If participants responded ‘No’ or ‘Unsure’ to question #1, they subsequently answered question #2, while bypassing questions 1a and 1b.

Participants then answered a series of bespoke questions probing their knowledge and understanding of MI, as well as their prior use of MI (see Fig 1 for a flowchart that illustrates the order of the questions). Participants also watched a brief informational video on what MI means to some people (see video from: https://osf.io/vky9d). The informational video included a definition of MI, a description of first-person (i.e., internal/egocentric perspective) and third-person (i.e., external/allocentric) visual MI, an explanation of how MI can support physical practice, and the use of MI in different contexts such as sports, music, arts, and activities of daily life, with examples for each.

Participants then completed a series of questionnaires, starting with an online version of the Kinesthetic and Visual Imagery Questionnaire [KVIQ; 38]. For the purpose of the current study, the short version of the KVIQ was administered such that participants’ imagery abilities were assessed on five different movements. For each movement, participants observed a short video clip of a female model performing a simple movement while seated (e.g., lifting non-dominant arm in air and returning it back down onto the lap); the video was viewed from the front with the model facing the participant (see pre-registration link for details: http://osf.io/26azs). Participants watched the video once and were then instructed to physically perform the same movement they had just observed in the video, one time. Subsequently, they imagined themselves performing the movement from a first-person (i.e., internal/egocentric) perspective, only one time, while concentrating primarily on the clarity of the image (i.e., visual imagery) before indicating the quality of their imagined movement on a five-point scale from 1 (“*no image*”) to 5 (“*image as clear as seeing*”). Participants imagined performing this same movement once more, but while focusing on the sensations and feelings of the imagined movement (i.e., kinesthetic imagery) before indicating the intensity of the sensations throughout their imagined movement on a five-point scale from 1 (“*no sensation*”) to 5 (“*as intense as executing the action*”). Participants completed this same procedure for each of the five movements. Notably, however, after reporting upon their kinesthetic imagery experiences for the first movement (i.e., lifting and lowering non-dominant arm), participants responded to an additional set of questions probing their imagery experiences. They were asked to describe what they saw/felt when imagining performing the first movement. The instructions for the first movement were as follows: “*Lift your non-dominant arm straight out in front of you and keep lifting until it is straight up high. Now return to the start position*.” Participants were asked to “*Describe what you saw when you imagined doing the movement (e.g., did you imagine seeing a certain side of your hand when you imagined performing the movement)?*”, and “*Describe what sensations you felt when you imagined doing the movement (e.g., did you imagine feeling a certain sensation on your hand)?*”. These follow up questions in which participants were asked to describe what they saw/felt while imagining the first movement on the KVIQ are denoted in Fig 1 by yellow stars numbered 2 and 3. All instructions were delivered to the participants in written format.

After finishing the KVIQ, participants completed the Full Scale Intelligence Quotient-2, which included the Vocabulary and Matrix Reasoning subscales of the Wechsler Abbreviated Scale of Intelligence [WASI-II; 41], the SRS-2 questionnaire [42] to measure social characteristics, and the ADC checklist [43] to measure motor ability (see Fig 1 for flowchart). Once completed, participants were sent a second link via Zoom to the behavioural experimental tasks that are reported in a separate paper [39].

### Data analysis

#### Bespoke survey questions.

To assess group differences in baseline knowledge of MI, prior experience with MI, and understanding of MI after watching the informational video, frequency data from five survey questions were analyzed. The five survey questions that were analyzed are denoted by a blue star in Fig 1. The traditional Chi-square test was not suitable for these data due to a violation of the expected frequency assumption – the value of the cell *expected* should be 5 or more in at least 80% of the cells [45]. As a result, these survey data were analyzed using five separate maximum likelihood ratio (MLR) Chi-square tests, which is a suitable alternative for smaller data sets that do not meet Chi-square assumptions [45]. The use of the MLR Chi-square tests allowed for a comparison of response distributions between autistic and non-autistic participants across each of the five survey questions, despite the small cell sizes.

#### Thematic analyses.

Thematic analyses were performed on three main qualitative data sets in this paper. The first set of data explored what participants thought MI might mean prior to watching the informational video describing MI. These data were analyzed thematically because understanding participants’ initial conceptualizations of MI is essential for assessing baseline knowledge. These data were particularly important to examine given that individuals may have different subjective understandings of the concept of MI, or no understanding of MI at all. If MI is to be used as a method to improve motor coordination difficulties in autistic individuals, it is important to first identify these baseline measures to help inform how educational materials and interventions are structured in the future. The second data set examined participants’ subjective experiences with visual imagery during the KVIQ, and the third data set examined participants’ subjective experiences with kinesthetic imagery during the KVIQ. The thematic analyses were performed by MB (a Ph.D. student in the field of motor control with experience in motor imagery research) and EG (a researcher with training in qualitative and quantitative methods and expertise in the field of motor imagery and motor control in autism). The survey data were saved and stored for offline analysis.

The researchers (MB and EG) followed Braun and Clarke’s [46–48] six-step process for reflexive thematic analysis to analyze the data, taking an inductive, semantic, and realist approach. Thematic analysis was utilized to enable the division of data into meaningful themes; this qualitative approach allows for comprehensive descriptions of data sets rather than mere data summarization. In addition, Braun and Clarke’s [46] approach is flexible and may be adapted to different research questions. As a result, MB and EG’s approach to the data was free from pre-existing theoretical assumptions/frameworks or pre-determined categories, to foster a genuine understanding of participants’ experiences from their own perspective.

The data were analyzed separately, such that EG familiarized herself with the first data set on what participants thought MI might mean, while at the same time MB familiarized herself with the second and third data sets on participants’ subjective visual and kinesthetic MI experiences with the KVIQ. EG and MB both read through their respective data sets whilst making any initial notes of key ideas or concepts. The second phase involved re-reading participants’ answers and identifying relevant codes relating to the scope of the study. Because the data were collected and stored online, all notes and coding processes were completed online in Excel. Both researchers differentiated autistic and non-autistic participant data by colour-coding. For the third phase, the codes were organized to form initial themes. This process involved grouping the different codes together based on similarity of content. Once no new themes could be developed, EG and MB reviewed each other’s codes and initial themes separately to recognize and add additional codes. In the fourth phase, EG and MB reviewed the data sets together to discuss the final set of codes and any changes made to the initial code names and themes. Some initial themes were merged because they were either too small on their own or described closely related codes. As a result, some initial themes were condensed into sub-themes. The themes were then discussed with the whole research team, where it was agreed that the codes and themes adequately represented the data. In the fifth phase, EG and MB allocated appropriate theme and sub-themes names, before commencing the sixth phase of writing the themes and supporting data into a report.

### Kinesthetic and visual imagery questionnaire (KVIQ)

The mean data from the KVIQ were then submitted to a Group (autistic, non-autistic) x Imagery Type (visual, kinesthetic) repeated measures analysis of variance (ANOVA). Partial eta squared values are reported as measures of effect size. Separate correlational analyses were also performed between participants’ average scores on the VI and KI components of the KVIQ with participants’ average scores on the Adult Developmental Coordination Disorder/Dyspraxia Checklist (ADC).

## Results

### Bespoke survey questions

**Fig 2:**
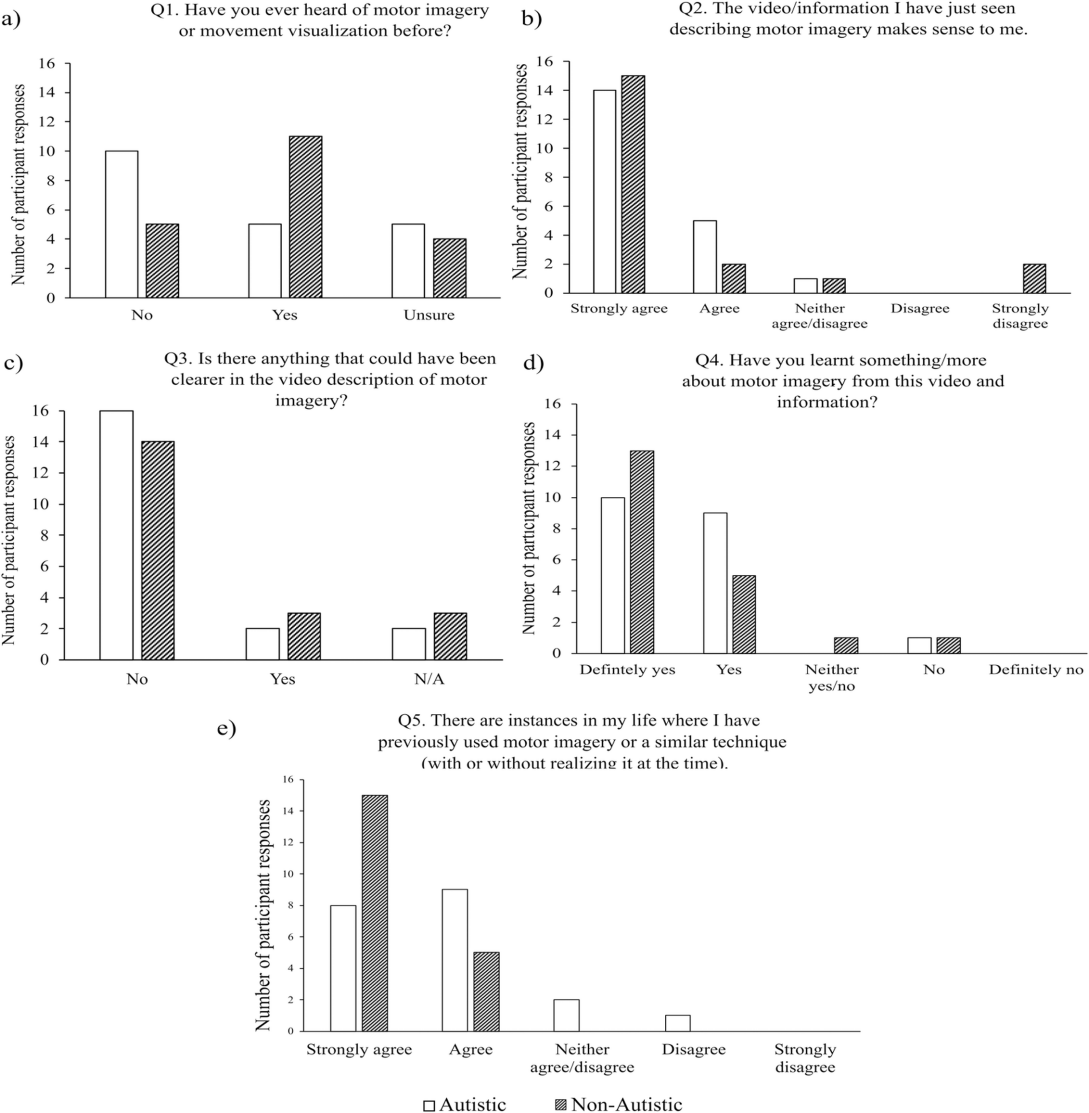
Participant Responses to MI knowledge and use. Number of participant responses for questions a) one, b) two, c) three, d) four, and e) five, probing participants’ knowledge/understanding of MI as well as their prior use of MI. Open bars represent the autistic group and dashed grey bars represent the non-autistic group.

Data from the five MI survey questions are presented graphically in Fig 2, with the results of the statistical analyses highlighted in Table 2. The results from the Maximum Likelihood Ratio (MLR) Chi-square tests indicated no statistically significant differences between the autistic and non-autistic participants for question one (see Fig 2a), question two (see Fig 2b), question three (see Fig 2c), or question 4 (see Fig 2d). However, two participants from the autistic group and 3 participants from the non-autistic group responded ‘yes’ to question 3, indicating that they felt the informational video lacked clarity in the description of MI. Notably, there was no overlap in participants who responded ‘yes’ to question 3 (i.e., “Is there anything that could have been made clearer in the video description of motor imagery?”), and who responded “Strongly disagree” to question 2 (i.e., “The video/information I have just seen describing motor imagery makes sense to me”). Some participants explained that the video could use “*more day-to-day examples in case you don’t play sports/music/dance*” (P36; non-autistic), or generally, “*more links to daily life*” (P5; autistic). Lastly, there was a trend for non-autistic individuals to report more prior use of MI than autistic individuals for question 5, but the MLR Chi-square test did not reach conventional levels of statistical significance (*p *= 0.06; see Fig 2e).

**Table 2:** MLR Chi-Square Analyses of MI knowledge and experience across groups.

Question	Response Option	Participant Group	Percentage Frequency (%)	Observed Count	Expected Count	MLR Chi-square (X^2^)	p-value	Effect Size (Cramer’s V)
Q1. “Have you ever heard of motor imagery or movement visualization before?”	Yes	Autistic	25%	5	8	X^2 ^= 4.12	*p* = 0.13	V = .317
		Non-Autistic	55%	11	8			
	No	Autistic	50%	10	7.5			
		Non-Autistic	25%	5	7.5			
	Maybe	Autistic	25%	5	4.5			
		Non-Autistic	20%	4	4.5			
Q2. “The video/information I have just seen describing motor imagery makes sense to me.”	Strongly Agree	Autistic	70%	14	14.5	X^2 ^= 1.62	*p* = 0.45	V = .198
		Non-Autistic	75%	15	14.5			
	Agree	Autistic	25%	5	3.5			
		Non-Autistic	10%	2	3.5			
	Neither agree/disagree	Autistic	0%	0	–			
		Non-Autistic	0%	0	–			
	Disagree	Autistic	5%	1	1			
		Non-Autistic	5%	1	1			
	Strongly Disagree	Autistic	0%	0	1			
		Non-Autistic	10%	2	1			
Q3. “Is there anything that could have been clearer in the video description of motor imagery?”	Yes	Autistic	10%	2	2.5	X^2 ^= 0.54	*p* = 0.77	V = .115
		Non-Autistic	15%	3	2.5			
	No	Autistic	80%	16	15			
		Non-Autistic	70%	14	15			
	N/A	Autistic	10%	2	2.5			
		Non-Autistic	15%	3	2.5			
Q4. “Have you learnt something/more about motor imagery from this video and information?”	Definitely yes	Autistic	50%	10	11.5	X^2 ^= 2.94	*p* = 0.40	V = .252
		Non-Autistic	65%	13	11.5			
	Yes	Autistic	45%	9	7			
		Non-Autistic	25%	5	7			
	Neither yes/no	Autistic	0%	0	0.5			
		Non-Autistic	5%	1	0.5			
	No	Autistic	5%	1	1			
		Non-Autistic	5%	1	1			
	Definitely No	Autistic	0%	0	–			
		Non-Autistic	0%	0	–			
Q5. “There are instances in my life where I have previously used motor imagery or a similar technique (with or without realizing at the time).”	Strongly Agree	Autistic	40%	8	11.5	X^2 ^= 7.48	*p* = 0.06	V = .396
		Non-Autistic	75%	15	11.5			
	Agree	Autistic	45%	9	7			
		Non-Autistic	25%	5	7			
	Neither agree/disagree	Autistic	10%	2	1			
		Non-Autistic	0%	0	1			
	Disagree	Autistic	5%	1	0.5			
		Non-Autistic	0%	0	0.5			
	Strongly disagree	Autistic	0%	0	–			
		Non-Autistic	0%	0	–			

MLR Chi-square analyses examining response distributions across autistic and non-autistic participants on five survey questions, probing baseline knowledge of MI, prior experience with MI, and their understanding of MI after watching the informational video.

### Thematic analysis

The thematic analyses are separated based on each of the three data sets. An overview of the themes (and sub-themes) can be found in Table 3. The full list of codes corresponding to each theme (and sub-themes) is provided in Supplementary S1-S3 Tables.

**Table 3:** Summary of themes, definitions, and sub-themes by question.

What do you think motor imagery might mean? (If you haven’t heard of motor imagery before, please give your best guess. If you really have no idea, please write “I don’t know”).		
**Themes**	**Theme definition**	**Sub-themes**
1. Imagination of Movement	General understanding of motor imagery	–
2. Non-Motor Imagery Concepts	Incorrect or lack of understanding of motor imagery	2.1 Vision-related 2.2 Non-specific ideas 2.3 Uncertain and not known
**Describe what you saw when you imagined doing the movement (e.g., did you imagine seeing a certain side of your hand when you imagined performing the movement?)**		
3. Experiences in Visual Motor Imagery	Participants’ visual-related experiences during visual motor imagery	3.1 Core visual imagery experiences 3.2 Effortful experiences 3.3 Experiences with imagery perspectives
4. Non-Visual Motor Imagery Experiences	Other non-visual experiences reported by participants	4.1 Kinesthetic motor imagery 4.2No imagery
**Describe what sensations you felt when you imagined doing the movement (e.g., did you imagine feeling a certain sensation on your hand?)**		
5. Experiences in Kinesthetic Motor Imagery	Participants’ kinesthetic-related experiences during kinesthetic motor imagery	5.1 Core kinesthetic imagery experiences 5.2 Emotional experiences 5.3 Experiences relating to movement planning
6. Non-Kinesthetic Motor Imagery Experiences	Other non-kinesthetic experiences reported by participants	6.1 Visual motor imagery 6.2 Other imagery experiences 6.3 No imagery

A summary of the six themes along with their definitions and sub-themes (where appropriate), grouped according to the question with which they are associated.

***What do you think motor imagery might mean? (If you haven’t heard of motor imagery before, please give your best guess. If you really have no idea, please write “I don’t know”).***

Two themes emerged from this data set: *Imagination of Movement* and *Non-Motor Imagery Concepts*. The second theme was supported by three sub-themes (Table 3).

### Theme 1: Imagination of movement

This theme represents those statements which indicate that the individual had a reasonable knowledge of what MI means and that MI involves imagining movement. Some participants gave more general responses, “*Thinking or imagining a movement or some kind of motor action*” (P29; non-autistic), while others gave a more specific definition that MI involved imagining their own body move, “*imagining a motor movement in your mind as though you were doing it yourself*” (P12; autistic). Some participants were highly accurate in their definition, including that MI involved both “*imagining doing a movement* *without moving yourself in real life*” (P7; autistic). This separation between imagining and executing was further highlighted by some participants describing MI as “*the co-ordination between how you imagine doing an action, and doing it*” (P20; autistic). Some participants also understood MI in terms of planning, “*It is the way you imagine movement and use when planning your movements*” (P25; non-autistic). There was an emphasis on visual MI with participants describing it as “*the ability to visualize movements*” (P11; autistic) or “*picturing the movements in my mind”* (P27; non-autistic).

### Theme 2: Non-motor imagery concepts

A number of participants did not have a clear idea of what MI means and their responses were broken into three sub-themes: 2.1) Vision-related, 2.2) Non-specific ideas, 2.3) Uncertain and not known.

#### 2.1 Vision-related.

In the first sub-theme, some participants thought that MI involved vision or visual images such as “*moving images*” (P13; autistic), “*Images relating to movement”* (P1; autistic), or *“images that require you to move your eyes*” (P34; non-autistic). One participant thought it involved other modalities too, “*Co-ordination of visual vs audio responses*” (P31; non-autistic).

#### 2.2 Non-specific ideas.

Participants in this theme provided general ideas around brain and motor processes, describing MI as “*the brain and the response to images*” (P26; non-autistic), “*how your motor skills work*” (P10; autistic), or “*imagination*” (P3; autistic).

#### 2.3 Uncertain and not known.

Some participants expressed uncertainty with the use of question marks in their responses, “*How you see?*” (P2; autistic), while others simply reported “*I don’t know*” (P21; non-autistic).

*Describe what you saw when you imagined doing the movement (e.g., did you imagine seeing a certain side of your hand when you imagined performing the movement?).*

A total of 2 themes were allocated to this data set with the first theme (*Experiences in Visual Motor Imagery*) broken down into 3 sub-themes and the second theme (*Experience with Imagery Perspectives*) broken down into 2 sub-themes.

### Theme 3: Experiences in visual motor imagery

Participants reported a variety of experiences during visual motor imagery and the responses were divided into three sub-themes: 3.1) Core visual imagery experiences, 3.2) Effortful experiences, and 3.3) Experiences with imagery perspectives.

#### 3.1 Core visual imagery experiences.

In this first sub-theme, some participants reported clear detailed images of themselves performing the movement, “*I saw I lift my left arm straight and rose it high. And then I put it back to the starting position*” (P39; non-autistic), “*I imagined myself performing the movement in the same way I just did, from the same angle as the video*” (P11; autistic). Others reported clear images, but with less detail, “*Yes, I can see myself performing the action*” (P38; non-autistic), “*I could see in my head lifting my arm up clearly*” (P17; autistic). Conversely, some participants reported a lack of clarity or blurred images during the imagery task, “*I imagined the entire arm moving but it was not extremely clear*” (P25; non-autistic), “*I saw my left arm and the back of my left hand moving up and down but it was blurry…*” (P36; non-autistic). Further, various participants explain how they could only imagine certain parts of the movement, “*Saw the initial lifting of the hand and the final putting it back down to rest on my thigh by (sic) not the middle of the movement*” (P8; autistic), “*The movement was not continuous*” (P33; non-autistic), while others reported imagining only certain body parts throughout the movement, “*Seeing upper side of my left hand*” (P37; non-autistic), “*I see the top side of my hands going upwards*” (P15; autistic).

#### 3.2 Effortful experiences.

One participant described their challenges with visual motor imagery by expressing the effort involved in imagining themselves performing the movement, “*If I force myself to imagine the action I can, but it is work*” (P16; autistic).

#### 3.3 Experiences with imagery perspectives.

Several visual perspectives were reported by participants, “*I could see myself from 3*^*rd*^ *person doing the movement as I closed my eyes*”, (P30; non-autistic), “*I saw myself performing the action from birds eye view*” (P32; non-autistic). Interestingly, one participant reported visualizing the movement from multiple perspectives at the same time, “*Two perspectives at once: One where I was completing the action in my own body, and another where I was sitting in the same way as the video displayed (3*^*rd*^ *person)*” (P7; autistic). Some participants also reported visualizing the model performing the movement rather than themselves, “*I imagined the person in the video raising her arm out, pausing then raising it up and back down*” (P6; autistic).

### Theme 4: Non-visual motor imagery experiences

Various participants reported the inability to engage in visual motor imagery or non-visual experiences during the visual imagery task. These responses are broken down into two sub-themes: 4.1) Kinesthetic motor imagery, and 4.2) No imagery.

#### 4.1 Kinesthetic motor imagery.

Interestingly, some participants reported the use of kinesthetic MI alongside vision during the visual MI task, “*imagined how it felt and what I saw whilst I was performing the movement*” (P40; non-autistic), “*just imagined the feeling of it more than the image, but saw the whole room and my own perspective when doing it”* (P12; autistic).

#### 4.2 No imagery.

Some participants shared that they visualized “*Nothing*” (P13; autistic), or that they “*Did not see an image*” (P14; autistic). Participant 5 (autistic) noted they could visually imagine the model presented in the video but could not imagine any movement, “*I saw the lady sitting in a chair. No movement*”.

*Describe what sensations you felt when you imagined doing the movement (e.g., did you imagine feeling a certain sensation on your hand?).*

Two main themes were applied to this data set, both organized into three sub-themes.

### Theme 5: Experiences in kinesthetic motor imagery

Participants reported a variety of experiences during kinesthetic motor imagery. The responses were divided into three sub-themes: 5.1) Core kinesthetic imagery experiences, 5.2) Emotional experiences, and 5.3) Experiences relating to movement planning.

#### 5.1 Core kinesthetic imagery experiences.

Many participants expressed sensing their muscles moving/contracting whilst imagining the movement, “*I felt the arm moving up, the muscles holding it up and I felt a strain on my arm*” (P21; non-autistic), “*I actually felt my muscles tense up*” (P19; autistic), while others explained that they could engage in kinesthetic imagery with less detail, “*Yes, I can feel my movement with imagination*” (P38; non-autistic). A tingle sensation was also reported by some participants, “*I felt a tingle on my non dominant arm*” (P23; non-autistic), or they imagined feeling the weight of their arm being lifted, “*feeling weight/pressure on my left hand*” (P37; non-autistic), “*I was trying to focus on what the weight of arm would feel like raising it up*” (P6; autistic). Furthermore, some participants described the feeling of air on their body as they imagined themselves moving, “*The feel of my arm cutting through the air*” (P32; non-autistic), or a stretching sensation, “*I imagined a stretching sensation in my shoulder*” (P16; autistic). Also, some participants specifically described where in their body (e.g., hand, arm) they felt the imagined sensations, “*I imagined the stretching motion of my hand”* (P35; non-autistic), “*I imagined a slight up and down motion in the arm*” (P13; autistic), *“I imagined the feeling of my arm doing the movement*” (P40; non-autistic).

#### 5.2 Emotional experiences.

In this sub-theme, some autistic participants described negative emotions/feelings associated with the kinesthetic imagery task. For instance, one participant reported they were “*Frustrated as I cannot imagine this…*” (P5; autistic), while another participant expressed that they felt “*a little nervous…*” (P4; autistic).

#### 5.3 Experiences relating to movement planning.

A few non-autistic participants referred to movement planning processes in their experiences with kinesthetic motor imagery, “*Hand wants to start moving upwards*” (P26; non-autistic), “*Hand and arm feel like they’re tingling as if the muscles are preparing to contract in the right way needed to do the movement*” (P28; non-autistic).

### Theme 6: Non-kinesthetic motor imagery experiences

A number of participants expressed non-kinesthetic related experiences whilst attempting to engage in kinesthetic motor imagery. The responses are organized into three sub-themes: 6.1) Visual motor imagery, 6.2) Other imagery experiences, and 6.3) No imagery.

#### 6.1 Visual motor imagery.

It was reported by some participants that they engaged in visual motor imagery during the kinesthetic motor imagery task, “*No real feeling just perfect vision of it happening*” (P31; non-autistic).

#### 6.2 Other imagery experiences.

In this sub-theme, some participants explained how they imagined the feeling of their clothing throughout the imagery task, “*I could feel my trousers...*” (P2; autistic), “*I imagine feeling the arm brushing against my shirt as I move it up*” (P30; non-autistic). Other participants reported imagining auditory sensations, “*I imagined the sound my chair makes and the noise my clothes make when they were moving…”* (P7; autistic), *“None – I was focused on listening*” (P3; autistic).

#### 6.3 No imagery.

Several participants had difficulty imagining the feelings and sensations associated with performing the movement. Some participants reported the inability to imagine the sensations by reporting “*No sensation*” (P11; autistic), or *“[I] didn’t feel any sensations in hand/arm*” (P8; autistic), while others noted the kinesthetic imagery task was more difficult than the visual imagery task, “*I couldn’t really imagine this because it isn’t visual*” (P18; autistic), or “*No real feeling just perfect vision of it happening*” (P31; non-autistic).

### Kinesthetic and visual imagery questionnaire (KVIQ)

**Fig 3:**
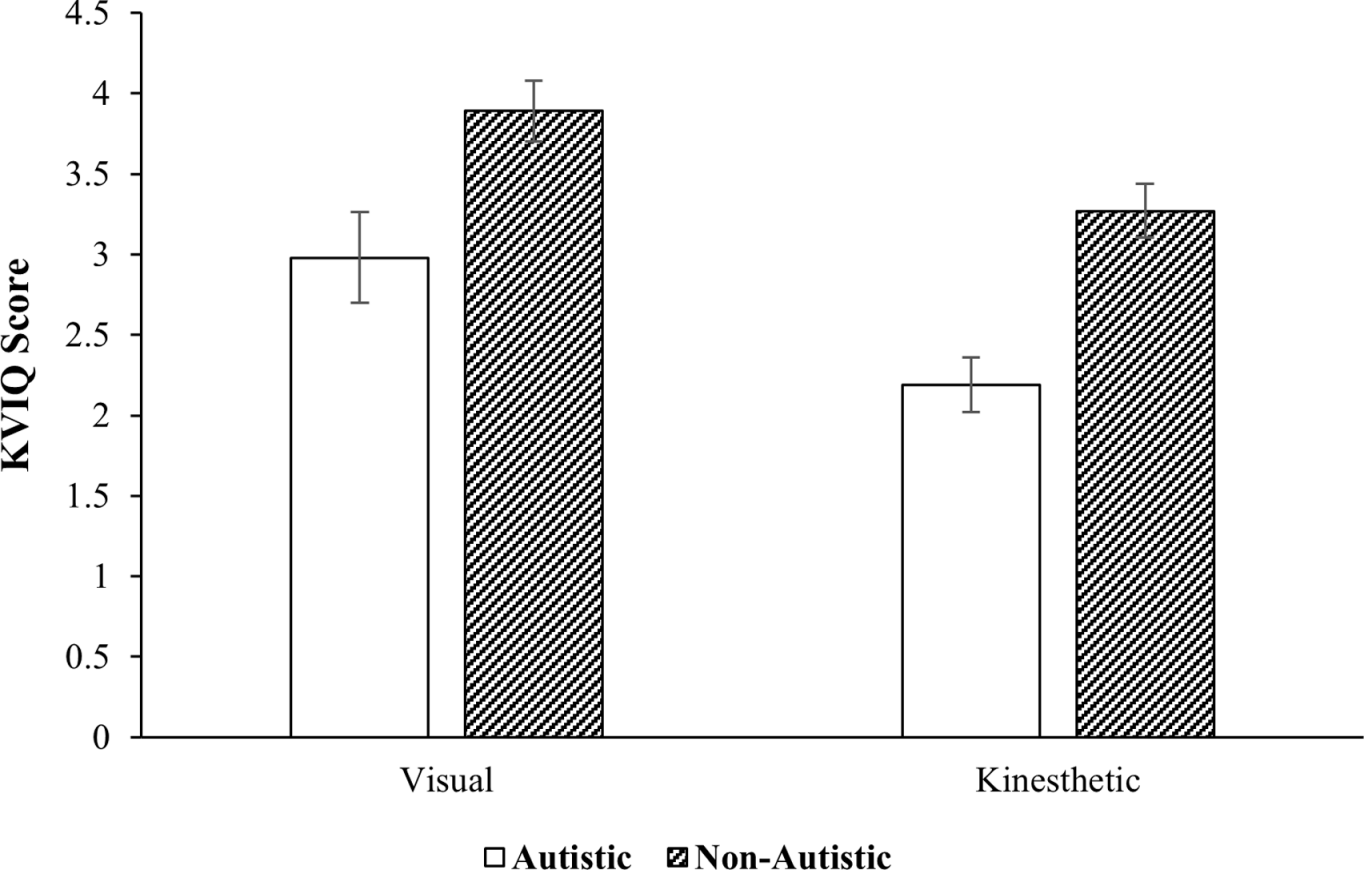
Mean KVIQ scores by group. Mean group scores from the Visual and Kinesthetic components of the KVIQ for the autistic and non-autistic participants. The participants reported their imagery quality on separate five-point scales ranging from 1 (“no image/no sensation”) to 5 (“image as clear as seeing/as intense as executing the action”). Open bars represent the autistic group and dashed grey bars represent the non-autistic group. Error bars represent standard error of the mean.

The results from the ANOVA showed a significant main effect of Group, *F*(1, 38) = 17.24, *p* < .001, $\eta_{p}$^2 ^= .312, indicating higher scores (i.e., clearer/more intense imagery) for the non-autistic group (*M* = 3.58, *SD* = 1.00) compared to the autistic group (*M* = 2.59, *SD* = 1.25). The results further showed a significant main effect of Imagery Type, *F*(1, 38) = 17.25, *p* < .001, $\eta_{p}$^2 ^= .312, where participants reported clearer/more intense imagery during visual imagery (*M* = 3.44, *SD* = 1.16) compared to kinesthetic imagery (*M* = 2.73, *SD* = 0.92). The Group x Imagery Type interaction was not significant, *F*(1, 38) = 0.25, *p* = 0.62, $\eta_{p}$^2 ^= .007. Mean data from the KVIQ are presented in Fig 3. Moreover, on an individual level, it is interesting to note that six autistic participants (P2, P3, P5, P13, P14, and P20) and zero non-autistic participants reported ‘No image’ during VI on at least one of the five movements, while ten autistic participants (P2, P3, P5, P6, P7, P8, P10, P11, P15, P18) and one non-autistic participant (P22) reported ‘No sensation’ during KI on at least one of the five movements. Further, one autistic participant (P3) and zero non-autistic participants reported no VI or KI on all five movements from the KVIQ, while two autistic participants (P13 and P20) and zero non-autistic participants reported no VI on all five movements.

The analyses of the relationship between KVIQ and ADC scores across all participants (regardless of group) revealed statistically significant negative correlations between mean VI scores and ADC total scores, VI: *r*(38) = −.363, *p *= .021, and between mean KI scores and ADC total scores, KI: *r*(38) = −.425, *p *= .006. These correlations reveal that, overall, lower scores on the subjective ratings of visual and kinesthetic imagery were associated with higher indices of developmental coordination disorder or dyspraxia (higher ADC scores) and vice versa.

## Discussion

The overall purpose of the current study was to describe the experiences of MI from the perspective of autistic adults. To the authors’ knowledge, this study is the first time that explicit MI was examined in autistic individuals using mixed-methods. The present study builds on previous investigations of MI in autism that have yielded varying patterns of findings [27,35,36]. The results from the present study first showed that autistic and non-autistic participants reported similar levels of knowledge about MI. There was also a trend for non-autistic participants to report more life experiences using MI prior to participating in the study compared to autistic participants, although the finding was not statistically significant. Thematic analyses revealed insight into the diverse experiences of both autistic and non-autistic participants during the VI and KI portions of the KVIQ, with responses ranging from vivid/intense images/sensations to the inability to imagine. Both autistic and non-autistic participants also reported a variety of imagery strategies. Further, results from the quantitative analysis of the KVIQ showed that autistic participants reported significantly less clear/intense imagery than their non-autistic counterparts, and that both groups reported their kinesthetic imagery (KI) as less clear than their visual imagery (VI). Together, the findings indicate that some autistic people have the capacity to engage in explicit MI, but like their non-autistic counterparts, there are individual differences in their MI abilities and subjective experiences.

At a group level, the autistic and non-autistic participants reported comparable knowledge of MI terminology prior to participating in the study. Some participants reported familiarity with the terms ‘motor imagery’ or ‘movement visualization’, while others had not encountered the terms before, and there was also a separate subset of participants who expressed uncertainty about the terms. The results from this particular question were helpful in assessing participants’ baseline knowledge of MI, and have important implications for MI interventions. That is, the results emphasize the importance of ensuring participants have sufficient knowledge of MI before engaging in an intervention because not everyone is initially aware of certain concepts or terms [40,49]. Recall, participants observed a brief informational video explaining the concept of MI to help familiarize them with the study’s context. The majority of participants (both autistic and non-autistic) understood the contents of the video, suggesting that the video could serve as an effective educational tool, especially with some changes and updates like additional examples. The dynamic and multi-sensory nature of the MI video may offer clearer explanations or demonstrations compared to written descriptions, making the concept potentially more comprehensible for a greater number of individuals. The fact that both groups found the video informative and clear also suggests its potential as a means for delivering MI instructions or educational resources within an intervention [see 50]. MI may be performed from different perspectives (i.e., first person vs. third person) and through different modalities (i.e., visual vs. kinesthetic), and thus it is important for interventions to offer clear and consistent instructions to reduce varied interpretations [40] – the results from the current study indicate that instructional videos may be an effective tool to achieve such clarity.

There was a trend in the data to suggest that, compared to the autistic participants, the non-autistic participants may have had more experience using MI in their everyday lives prior to their participation in the study. This finding could be attributed to differences in imagery (in)ability or perhaps exposure to contexts in which MI may be used such as athletics or the performing arts [see 51]. Previous work by Gowen and colleagues [5] showed that autistic adults reported that their reduced motor abilities have led them to avoid certain sporting and/or social situations due to a combination of physical and mental effort required, as well as a fear of failure, bullying, and embarrassment. The avoidance of such situations may then reduce their overall experiences with MI. It is also possible that autistic people use MI in their daily lives as much as non-autistic people, they just may not realize it or recognize it as such.

Responses to the open-ended questions from the KVIQ suggest that some autistic people can engage in MI, while others cannot [see 52 for related findings on visualization challenges]. Like their non-autistic counterparts, autistic adults seem to differ from one another in terms of their MI abilities and the strategies they use which has implications for MI interventions. MI may be used as a method to improve motor coordination in autistic adults, yet the pattern of findings emphasizes the need to screen autistic adults on an individual basis to determine who may benefit from such an intervention. More specifically, although MI may be a promising tool for improving motor coordination in autistic adults, its success will depend on understanding and considering an individual’s ability to effectively engage in MI (visually and/or kinesthetically). Moreover, the results demonstrated a variety of strategies used by both autistic and non-autistic participants. For example, during VI, some participants reported imagining from different visual perspectives, such as a first- or third-person perspective. In fact, one autistic participant reported imagining the movement from both perspectives at the same time. Also during VI, there were reports of participants engaging in KI at the same time, presumably as a strategy to reinforce their VI. For the KI portion of the questionnaire, some participants reported using tactile, auditory, or proprioceptive strategies. They described imagining the feeling of their clothing, the sounds associated with the movement, or the sensation of their muscles stretching whilst imagining the feeling of performing the movement. Imagining different sensory modalities during KI could be a helpful strategy for some individuals to aid the imagined sensations of the movement. Overall, the responses indicate that non-autistic and some autistic participants were able to engage in VI and KI, but varied in the strategies they used and the extent to which they could imagine themselves moving. Critically, the findings from the open-ended KVIQ questions indicate varied interpretations of the instructions. For instance, some participants explained how they imagined the model in the demonstration video performing the movements instead of themselves, despite the initial instructions to “…imagine yourself doing the movement (without actually doing it – just imagining)”. Less specific instructions throughout the questionnaires, such as, “Now imagine the movement you just practiced” may have caused confusion about whether to imagine themselves or the model in the demonstration video performing the movement. The VI instructions also did not describe a specific perspective for participants to imagine from (i.e., first or third person), and so it is not surprising that there were reports of engaging in MI from different visual perspectives. These discrepancies in the interpretation of the instructions led some participants to report inconsistent answers on the KVIQ. For example, a participant rated their imagery as ‘clear’ but then described that they imagined the model in the video performing the movement, not themselves. Relying solely on the quantitative data may inaccurately suggest that this participant is proficient at MI, despite the fact that they could not (or chose not to) imagine *themselves* moving. These data underscore the importance of clear, consistent instructions to ensure accurate participant interpretation, as failure to do so may introduce variability in the data and undermine the integrity of the findings [40]. Certain methodological improvements should be considered with future use of the KVIQ in online settings, such as clear and detailed instructions, along with supervised sessions where participants are asked to repeat the instructions back to the researcher to ensure comprehension.

The quantitative analysis of the KVIQ indicates that on a group level, the autistic participants reported less vivid imagery than the non-autistic participants. This pattern of results may stem from differences in motor simulation processes between autistic and non-autistic individuals, and/or related to the motor execution difficulties experienced by autistic individuals. Past work has shown that autistic individuals experience difficulties with imitation [4,24], action perception [39], action understanding [23], and action prediction [21] – abilities which rely on motor simulation. Because motor simulation is also implicated in MI, the lower KVIQ scores reported by the autistic group in the current study could be attributed to altered simulation processes. The autistic participants also reported significantly higher total scores on the ADC, indicating they experience more difficulty with everyday movement-related tasks compared to the non-autistic participants. The fact that the VI and KI scores from the KVIQ were negatively correlated with participants’ total ADC scores suggests that participants who reported less clear/intense VI/KI also experienced more everyday movement difficulties. Higher scores on the ADC, and/or less prior experience using MI, could explain why the autistic participants reported lower scores on the KVIQ compared to non-autistic participants. The negative correlation between ADC and KVIQ scores is consistent with the notion based on ideomotor/common coding theory that the neural codes underlying movement execution are also engaged during MI. In other words, if the motor codes leading to movement execution are not sufficiently formed to support efficient action, then these codes might also lead to inefficient MI (or the variable MI is consistent with the variable motor codes for execution). Previous studies using explicit MI tasks [e.g., 35,36] have shown similar findings, where autistic participants were found to have poorer imagery compared to their non-autistic counterparts. Yet, the findings from the current study do not align with those from Gowen et al. [21] who found no significant group differences between autistic and non-autistic participants on the KVIQ (although there was a non-significant trend for autistic individuals having lower visual imagery scores than non-autistic individuals, as in the present study). Previous research has highlighted a considerable amount of inter-individual variability in MI ability in non-autistic populations, suggesting that individuals may differ in the way or the extent that they can imagine themselves moving [53–55]. This variability may be due to the multidimensionality of MI, wherein certain dimensions (i.e., motor image generation, manipulation, or maintenance) may be easier for some people than others [56,57]. The KVIQ primarily measures motor image generation (i.e., participants generate an image and rate how clear/intense it is) and thus may not provide a complete assessment of one’s MI abilities, as the manipulation and maintenance dimensions are left unexplored [58]. Although the demographics of the participants in the current study are similar to those of the participants in Gowen et al. [21], the heterogeneity within the autistic population may further compound the inter-individual variability in MI ability, offering a possible explanation for the discrepancies in the KVIQ results between the current study and Gowen et al. [21]. In addition, the KVIQ in the current study was administered in an online format, whereas the participants in [21] completed the KVIQ in-person. These methodological differences could further explain the discrepancies in the results, while highlighting the need for consistency between studies. Finally, the results from the current study showed that both the autistic and non-autistic groups reported more vivid imagery when imagining the movements visually (VI) compared to kinesthetically (KI). The directionality of these findings (i.e., higher VI scores, lower KI scores) is consistent with previous work [21,38] and suggests that VI is generally easier than KI for both non-autistic and autistic populations. Interestingly, several participants (autistic and non-autistic) reported that their initial understanding of MI was related to vision. This finding suggests that KI may have been an abstract or challenging concept for some individuals, potentially making it more difficult for them to perform.

In addition to the methodological limitations mentioned above, there are other important limitations that should also be addressed. First, although a researcher monitored participants throughout the study via video calls, inherent challenges associated with online studies remain. Specifically, there was a lack of control over the participants’ environments because participants were completing the tasks at home. This variability in environment could have introduced distractions for the participants, and the webcam setup made it difficult to view their entire surroundings and ensure they were performing the movements correctly and accurately. Second, participants were asked to describe what they saw or felt after only one movement on the KVIQ. Just as a participant’s ratings for clarity of image or intensity of sensation can vary from one movement to the next (i.e., rating the clarity a 4 on the first movement and then a 2 for the next), their subjective experiences may also fluctuate. Thus, by limiting our qualitative inquiry to a single movement, it is possible that we did not capture the full range of participant experiences throughout the entire KVIQ assessment. Furthermore, because the movements on the KVIQ are relatively simple, the assessments might not have been sensitive to differences in MI abilities between autistic and non-autistic adults that may be more pronounced with more complex actions. Although a self-report measure was critical to understanding the subjective experiences of the participants, there are potential biases involved, like social desirability and recall bias, that should be considered. Motor imagery was examined using a more objective measure, specifically mental chronometry (i.e., comparing the amount of time it takes to imagine a movement versus physically performing the same movement), as part of the larger research project, but these data are outside the scope of the current study [see 39 for the related paper that includes these objective MI measures]. Finally, the small sample size and variability should be considered when determining the applicability of the study’s results. That is, motor imagery ability can vary widely between individuals, and autism characteristics exist along a broad spectrum. Together, this variability means that caution may be needed when generalizing the findings to other individuals. Future MI research should incorporate both qualitative and quantitative measures to examine potential factors that contribute to individual differences in MI abilities and experiences among autistic adults.

The findings from this study have implications for the design of MI interventions for autistic adults. First, because the results reveal that not everyone is familiar with certain terminology or concepts (both autistic and non-autistic individuals), the findings underscore the importance of assessing baseline knowledge of MI before initiating any MI intervention. Given the mixed abilities of autistic individuals engaging in MI, clinicians and practitioners should also consider personalized screening to assess who may benefit from MI-based interventions. For example, the findings from this study indicate that individuals who report difficulty with MI may need more tailored support, such as using multisensory strategies or adjusting the level of complexity of the MI tasks. In addition, it is possible that clinicians may also benefit from using educational materials, such as the informational video used in this study, as part of an intervention to improve participant understanding and increase engagement. Furthermore, the observed variability in the MI strategies reported by participants in this study, such as tactile and auditory cues, offers a way for clinicians to incorporate flexible MI approaches that align with individual abilities or preferences. Finally, a key takeaway is the importance of clear, consistent instructions. Careful attention to how MI exercises are introduced and how feedback is provided can help ensure participants fully understand the tasks and perform them correctly. Importantly, the questions and procedures from the current experiment were pre-screened by a consultation group of autistic individuals to ensure that terminology and instructions were clear and accessible, reducing the variability in understanding between groups. Together, these strategies can help clinicians and practitioners administer more effective MI interventions for autistic adults, with the ultimate goal of improving motor coordination.

## Conclusion

This study was the first known study to take a mixed-methods approach to exploring explicit MI in autistic adults. The findings showed similarities and differences between autistic and non-autistic adults in their MI abilities and experiences. While the groups reported comparable MI knowledge, autistic participants tended to report less clear/intense imagery compared to their non-autistic counterparts. Thematic analyses offered valuable insights into the unique experiences and diverse strategies employed by participants across both groups during the KVIQ MI tasks. Considering the variability in MI abilities, direct comparisons between autistic and non-autistic populations at a group level remain challenging. Nevertheless, this study demonstrated that while some autistic individuals possess the capacity to engage in motor imagery, others do not. Therefore, while MI interventions may be useful for improving motor coordination in autistic adults, the findings underscore the need for individualized screening to ascertain an individual’s MI abilities beforehand. Overall, this study contributes significantly to the understanding of explicit MI processes in autistic adults, shedding light on the nuanced nature of simulation processes and highlighting the importance of methodological rigor in studying this population.

## Supporting information

Table S1 Motor Imagery Knowledge Codes. The codes associated with participants’ baseline knowledge of motor imagery.
(PDF)

Table S2 Visual Motor Imagery Codes. The codes associated with participants’ subjective experiences during visual motor imagery.
(PDF)

Table S3 Kinesthetic Motor Imagery Codes. The codes associated with participants’ subjective experiences during kinesthetic motor imagery.
(PDF)

Table S4 Motor Imagery GRASS Checklist Part A: Essential items for general study reporting. (PDF)

Table S5 Motor Imagery GRASS Checklist Part B: Essential items relating specifically to motor imagery. (PDF)

Table S6 Motor Imagery GRASS Checklist Part C: Discretionary Items. (PDF)
