# Supplementary material for: A mixed methods exploration of motor imagery in autistic and non-autistic adults: Diverse experiences and implications for interventions
Source: PLoS One. 2025 Jun 26;20(6):e0326542. doi: 10.1371/journal.pone.0326542 (PMC12200693; doi:10.1371/journal.pone.0326542)
Supplement: Table S1 — The codes associated with participants’ baseline knowledge of motor imagery. (PDF) [file pone.0326542.s001.pdf]

Table S1. Motor Imagery Knowledge Codes. The twenty-one codes associated with participants' baseline knowledge of motor imagery. The codes are grouped according to their assigned theme and sub-theme (where applicable), and based on the frequency of reports within autistic and non-autistic participants.

What do you think motor imagery might mean? (If you haven't heard of motor imagery before, please give your best guess. If you really have no idea, please write "I don't know").		
**Theme**		
	**Frequency by Group**	
**1. Imagination of Movement**	**Autistic**	**Non-Autistic**
1. Imagining movement	0	3
2. Using visualization to move the body	0	1
3. Visually imagining movement	1	3
4. Visually imagining yourself move	2	0
5. Imagining yourself move without actually moving	4	2
6. Imagining yourself move	5	2
7. Relationship between imagining and doing	1	1
8. Planning movement before doing it	2	4
**2. Non-Motor Imagery Concepts**		
2.1 Vision-related		
9. Vision	1	0
10. Visual images	1	1
11. Moving images	1	1
12. Perceiving moving things	0	1
13. Visual images of movement	1	0
14. Following images with your eye movements	0	1
15. Coordinating visual and auditory responses	0	1
16. Picture/visualization of body structures	0	1
2.2 Non-specific ideas		
17. Motor skills	1	0
18. Brain/cognitive process	2	1
19. Imagination	2	0
2.3 Uncertain and not known		

20. Uncertainty	3	2
21. Unknown	2	5
