# Supplementary material for: A mixed methods exploration of motor imagery in autistic and non-autistic adults: Diverse experiences and implications for interventions
Source: PLoS One. 2025 Jun 26;20(6):e0326542. doi: 10.1371/journal.pone.0326542 (PMC12200693; doi:10.1371/journal.pone.0326542)
Supplement: Table S2 — The codes associated with participants’ subjective experiences during visual motor imagery. (PDF) [file pone.0326542.s002.pdf]

Table S2. Visual Motor Imagery Codes. The twelve codes associated with participants' subjective experiences during visual motor imagery. The codes are grouped according to their assigned theme and sub-theme, and based on the frequency of reports within autistic and non-autistic participants.

Describe what you saw when you imagined doing the movement (e.g., did you imagine seeing a certain side of your hand when you imagined performing the movement?).		
**Theme**		
	**Frequency by Group**	
**1. Experiences in Visual Motor Imagery**	**Autistic**	**Non-Autistic**
3.1 Core visual imagery experiences		
1. Visually imagined themselves performing movement	8	14
2. Only imagined certain parts of the movement	1	1
3. Lack of clarity/blurry image	1	3
4. Imagined the hands	2	3
5. Imagined the arms	3	7
3.2 Effortful experiences		
6. Imagery was effortful	1	0
3.3 Experiences with imagery perspectives		
7. Imagined from 3rd person perspective	3	2
8. Imagined from a bird's eye view perspective	0	1
9. Imagined the model performing the movement	2	0
**2. Non-Visual Motor Imagery Experiences**		
4.1 Kinesthetic motor imagery		
10. Imagined the feeling of the movement	1	1
4.2 No imagery		
11. Imagined model but no movement	1	0
12. Could not visually imagine	3	0
