# Supplementary material for: A mixed methods exploration of motor imagery in autistic and non-autistic adults: Diverse experiences and implications for interventions
Source: PLoS One. 2025 Jun 26;20(6):e0326542. doi: 10.1371/journal.pone.0326542 (PMC12200693; doi:10.1371/journal.pone.0326542)
Supplement: Table S3 — The codes associated with participants’ subjective experiences during kinesthetic motor imagery. (PDF) [file pone.0326542.s003.pdf]

Table S3. Kinesthetic Motor Imagery Codes. The twenty-two codes associated with participants' subjective experiences during kinesthetic motor imagery. The codes are grouped according to their assigned theme and sub-theme, and based on the frequency of reports within autistic and non-autistic participants.

Describe what sensations you felt when you imagined doing the movement (e.g., did you imagine seeing a certain side of your hand when you imagined performing the movement?).		
**Theme**		
	**Frequency by Group**	
**1. Experiences in Kinesthetic Motor Imagery**	**Autistic**	**Non-Autistic**
5.1 Core Kinesthetic imagery experiences		
1. Mild/slight imagined sensation	4	2
2. Imagined tingling feeling	0	2
3. Imagined sensation of effort	1	3
4. Imagined sensation of weight	2	2
5. Kinesthetic imagery of hand	0	4
6. Kinesthetic imagery of arm	3	9
7. Kinesthetic imagery of shoulder	2	1
8. Imagined pain	0	1
9. Imagined proprioception	0	1
10. Imagined sensation of stretching	1	2
11. Kinesthetic motor imagery	5	6
12. Imagined touch sensation	2	0
13. Imagined feeling of air on body	0	2
14. Imagined muscles moving/contracting	1	3
15. Imagined arm feeling tired	0	1
5.2 Emotional experiences		
16. Feeling frustrated	1	0
17. Feeling nervous	1	0
5.3 Experiences related to movement planning		
18. Imagined movement planning	0	2
**2. Non-Kinesthetic Motor Imagery Experiences**		
6.1 Visual motor imagery		
19. Visual motor imagery	1	1

6.2 Other imagery experiences		
20. Imagined feeling of one's clothing	2	1
21. Imagined auditory sensation	2	1
6.3 No imagery		
22. No kinesthetic imagination	9	1
