# Supplementary material for: A mixed methods exploration of motor imagery in autistic and non-autistic adults: Diverse experiences and implications for interventions
Source: PLoS One. 2025 Jun 26;20(6):e0326542. doi: 10.1371/journal.pone.0326542 (PMC12200693; doi:10.1371/journal.pone.0326542)
Supplement: Table S4 — (PDF) [file pone.0326542.s004.pdf]

Table S4. Motor Imagery GRASS Checklist Part A: Essential items for general study reporting

	Item	Pages
A1	Are participant characteristics (age, sex, handedness, experience with similar tasks, vision, clinical details, etc) included in the final study sample/groups?	8-9
A2	What instructions were provided? How were they delivered (spoken, written, etc)?	10-11
A3	Were standard isntructions used (i.e., a script, information sheet etc?) Is this available to readers (in the manuscript, supplementary materials, an online repository, etc)?	10-11
A4	Was adherence to instructions monitored (e.g., EMG recordings, post test questionnaires, repeated instrcutions, manipulation checks, etc)?	11
A5	Do statistical comparisons include the average and standard deviation or standard error of the mean for the groups/conditions?	18
A6	Is the ‘dose’ used in the study cleary defined (i.e., sessions, blocks, trials, duration, etc)?	10-11
