# Supplementary material for: A mixed methods exploration of motor imagery in autistic and non-autistic adults: Diverse experiences and implications for interventions
Source: PLoS One. 2025 Jun 26;20(6):e0326542. doi: 10.1371/journal.pone.0326542 (PMC12200693; doi:10.1371/journal.pone.0326542)
Supplement: Table S5 — (PDF) [file pone.0326542.s005.pdf]

Table S5. Motor Imagery GRASS Checklist Part B: Essential items relating specifically to motor imagery

#	Item	Pages
B1	Were participants instructed to use kinesthetic imagery, visual imagery, or a combination of both?	10-11
B2	If visual imagery was used, is the visual perspective (1st person, 3rd person) stated?	-
B3	If 3rd person imagery was used, is the vantage point specified? Is it illustrated?	-
B4	Were participants previously familiar with motor imagery (e.g., sports practice, prior participation in experiments)?	10
