# Supplementary material for: A mixed methods exploration of motor imagery in autistic and non-autistic adults: Diverse experiences and implications for interventions
Source: PLoS One. 2025 Jun 26;20(6):e0326542. doi: 10.1371/journal.pone.0326542 (PMC12200693; doi:10.1371/journal.pone.0326542)
Supplement: Table S6 — (PDF) [file pone.0326542.s006.pdf]

Table S6. Motor Imagery GRASS Checklist Part C: Discretionary Items (to be included as appropriate on a case-by-case basis)

#	Item	Pages
C1	Are study materials/data/code openly available (inlcuding a link to a repository)?	8
C2	Were imagery instructions based on a framework (e.g., PETTLEP, LSRT)? If so, how?	-
C3	Was imagery ability/quality assessed (e.g., questionnaires, chronometry)?	10-11
C4	Was the participant's body posture matched with the action(s) they imagined (e.g., were imagined/actual postures matched, mirror, etc)?	10-11
C5	Was movement during imagery instructed/allowed (e.g., dynamic motor imagery)?	10-11
C6	Were other modalities of imagery (e.g., auditory, haptic, olfactory, gustatory) instructed or reported by participants?	27, 32
